# Improving the diagnostic performance of contrast-enhanced mammography through lesion conspicuity and enhancement quantification

**DOI:** 10.1007/s00330-025-11501-8

**Published:** 2025-04-03

**Authors:** Iris Allajbeu, Muzna Nanaa, Roido Manavaki, Vasiliki Papalouka, Ioana Bene, Nicholas Payne, Elisabetta Giannotti, Thiemo van Nijnatten, Fleur Kilburn-Toppin, Nuala Healy, Fiona Gilbert

**Affiliations:** 1 Department of Radiology, University of Cambridge School of Clinical Medicine, Cambridge, UK; 2 Department of Radiology, Addenbrookes Hospital, Cambridge University Hospitals NHS Foundation Trust, Cambridge, UK; 3 Western Balkans University, School of Clinical Medicine, Tirana, Albania; 4 Breast Unit, St Bartholomew’s Hospital, West Smithfield, London, UK; 5 ‘Iuliu Hatieganu’ University of Medicine and Pharmacy, Cluj-Napoca, Romania; 6 Department of Radiology and Nuclear Medicine, Maastricht University Medical Center+, Maastricht, The Netherlands; 7 GROW Research Institute for Oncology and Reproduction, Maastricht University, Maastricht, The Netherlands; 8 Medical Center+, Maastricht, The Netherlands; 9 Beaumont Breast Centre, Beaumont Hospital, Dublin, Ireland; 10 Department of Radiology, Royal College of Surgeons, Dublin, Ireland

**Keywords:** Contrast-enhanced mammography, Breast cancer, Conspicuity, Contrast, Enhancement intensity

## Abstract

**Objectives:**

To analyze qualitative and quantitative enhancement of breast lesions on CEM and their impact on specificity and overall diagnostic performance in predicting malignancy. A secondary objective was to compare lesion enhancement patterns between CEM and contrast-enhanced (CE)-MRI.

**Methods:**

The cohort included screening and symptomatic cases from CEM research studies (December 2016–March 2023) with an identifiable lesion. Three breast radiologists independently assessed lesion conspicuity as low, moderate, or high, based on the BI-RADS CEM lexicon. Lesion enhancement was quantified by drawing two regions of interest representing lesion and background parenchyma, to calculate contrast enhancement from the early (CE_early_) and late (CE_late_) views. Area-under-the-curve (AUC) was used to assess diagnostic performance, with thresholds determined using the maximum Youden index. Cohen’s κ was used to measure agreement between CEM and DCE-MRI enhancement patterns. *p*-values < 0.05 were deemed statistically significant.

**Results:**

From 503 CEM studies, 143 BI-RADS 2–5 lesions were analyzed. Lesion conspicuity was significantly associated with lesion histology (*p* < 0.001), contrast enhancement metrics (CE_early_, CE_late_), and enhancement patterns on CEM recombined images. CE_early_ performed better in differentiating malignant from benign lesions or background parenchymal enhancement (BPE), with AUC values of 0.83 and 0.88 and 90% specificity in distinguishing BPE from cancers. There was fair/moderate agreement between lesion enhancement patterns on CEM and DCE-MRI (Cohen’s κ = 0.35, *p* < 0.001), with a higher agreement for lesions exhibiting a wash-out pattern (Cohen’s κ = 0.5, *p* < 0.001).

**Conclusion:**

Both conspicuity and quantification of lesion enhancement can improve CEM specificity in predicting malignancy, with CE_early_ offering the best diagnostic performance.

**Key Points:**

***Question*** *Quantifying lesion enhancement conspicuity on contrast-enhanced mammography (CEM) has demonstrated potential in differentiating malignancy from benign lesions and BPE*.

***Finding*** *Contrast from the early recombined view (CEearly) performed better in discriminating malignant from benign lesions and BPE, with 90% specificity for BPE vs cancers*.

***Clinical relevance*** *Conspicuity and quantification of lesion enhancement on CEM can improve the specificity and overall diagnostic performance of CEM in cancer detection. Implementation of conspicuity thresholds in routine CEM interpretation could potentially reduce unnecessary recalls and benign biopsies*.

**Graphical Abstract:**

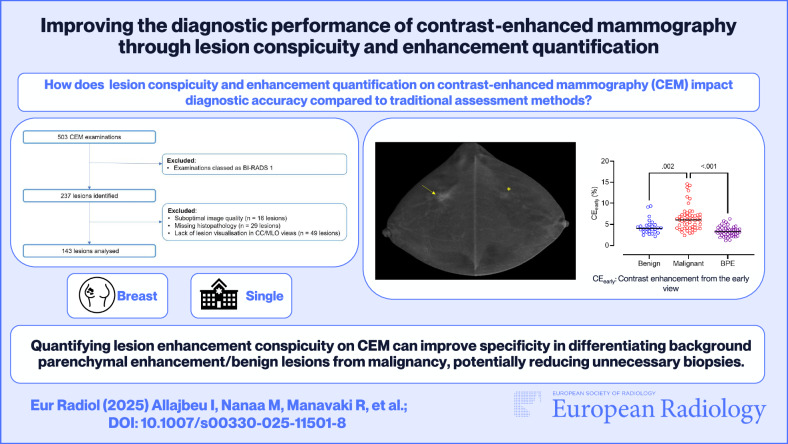

## Introduction

Contrast-enhanced mammography (CEM) is a functional imaging technique that has gained popularity in clinical practice recently. It is a dual-energy recombination method of two-dimensional images in both craniocaudal (CC) and mediolateral oblique (MLO) views following intravenous contrast administration to detect enhancing breast lesions. Like dynamic contrast-enhanced magnetic resonance imaging (DCE-MRI), CEM can provide information about lesion vascularity [1]. Lesion enhancement intensity and kinetics from DCE-MRI have shown utility in differentiating benign from malignant lesions, with strong enhancement and wash-out patterns being indicative of malignancy [2]. Furthermore, features from DCE-MRI can help differentiate tumor subtypes [3] and predict lymph node metastasis [4].

Published studies suggest that CEM has comparable sensitivity to MRI for the detection of breast cancer [1, 4–10]. However, one of the drawbacks of both MRI and CEM is the relatively low specificity that can lead to unnecessary recalls [5]. Qualitative and quantitative analysis of lesion enhancement on CEM has been investigated with promising but variable results [11–16]. A recent review of 23 studies quantifying lesion enhancement on CEM [17] highlighted a tendency for benign lesions to show weaker enhancement compared to malignancy. Additionally, two studies noted similar enhancement patterns between CEM and MRI, with malignant lesions mainly demonstrating wash-out enhancement, and benign lesions showing progressive enhancement patterns [13, 14].

Lesion conspicuity, a unique BI-RADS descriptor for CEM that has been recently included in the lexicon, has shown promise in predicting malignancy [18, 19]. Recent studies have tried to differentiate between invasive and non-invasive tumors or hormone receptor status based on lesion enhancement with variable results [13, 19]. Like DCE-MRI, differentiating background parenchymal enhancement (BPE) from actual breast lesions is another challenge with CEM [20, 21]. Quantitative methods might improve lesion characterization on CEM and guide clinical management.

This study aimed to assess the qualitative and quantitative enhancement of breast lesions on CEM recombined images, investigating their impact on specificity and overall diagnostic performance in predicting malignancy. A secondary objective was to correlate lesion enhancement patterns between CEM and MRI in a subgroup of patients who underwent both examinations.

## Materials and methods

The study cohort was created by selecting lesions from CEM examinations performed between December 2016 and March 2023 as part of two ethically approved prospective research studies in our institution. The first study (CONTEND; NCT02479100) was a prospective, single-center, randomized controlled pilot study carried out between 2016 and 2019, comparing usual care (mammography, ultrasound with/without MRI) and the addition of CEM for the pre-operative staging of breast cancer. The second study (BRAID; NCT04097366) is a recent prospective multicenter trial in the UK, comparing supplemental imaging with abbreviated breast MRI, automated breast ultrasound, or CEM for the detection of breast cancer in women aged 50–70 years with dense breasts. Participants in both studies provided informed consent.

### Inclusion/exclusion criteria

CEM examinations with BI-RADS 2–5 lesions that were either biopsy-proven or followed up for at least 2 years were eligible for inclusion. Exclusion criteria were suboptimal image quality, missing histopathology, or lack of lesion visualization on one or both views.

### Image acquisition

#### CEM

Examinations were performed on a senographe essential (GE Healthcare) or Pristina (GE Healthcare) digital mammography system. Both trials utilized similar CEM acquisition protocols. In brief, following screening for contraindications to contrast injection, 100 mL Iohexol 300 (Omnipaque, GE Healthcare) was administered intravenously with a power injector at 3 mL/s. A pair of low-energy (LE) and high-energy (HE) images were subsequently obtained in the CC and MLO views of each breast, starting at 2 min after contrast injection. Voltage settings depended on breast density and thickness as chosen by automatic exposure controls (26–31 kVp for LE exposures; 45–49 kVp for HE exposures). If a lesion was suspected, the breast of concern was imaged first. The time interval between early (CC) and late (MLO) views ranged between 4 min and 5 min. Recombined images for each view were generated by each system’s proprietary algorithm.

#### MRI

All breast MRI examinations were performed on an Optima MR450w 1.5-T MRI scanner (GE Healthcare) with a dedicated 8-channel breast coil, using a standard full clinical protocol. Following the acquisition of the localizer, a 2-mm axial T2-weighted sequence was obtained. DCE-MRI involved a 3D fat-saturated T1-weighted image (slice thickness: 2 mm), followed by five phases at 84 s intervals after administration of 0.1 mmol/kg Gadovist (Bayer Healthcare) at 2 mL/s via a pump and a 20 mL saline flush. Post-contrast subtracted images and maximum intensity projections were generated for review.

### Image analysis

Image analysis involved retrospective evaluation of prospectively acquired images, with all assessments conducted at our institution by readers who were blinded to the patient clinicopathological information. On CEM recombined images, an enhancing lesion was defined as an asymmetric area of enhancement similar to or stronger than BPE. Three radiologists, with 6 (I.B.), 7 (M.N.), and > 10 years (I.A.) of experience in breast imaging, and 2 years, 3 years, and 5 years of experience in CEM, respectively, independently assessed conspicuity for each lesion based on the latest BI-RADS CEM lexicon [18]. BPE was assessed in both CC and MLO views, with particular attention to focal asymmetric enhancement. According to the BI-RADS CEM lexicon [18], focal asymmetric BPE may mimic or obscure pathological lesions, necessitating further evaluation. This distinction is critical as focal asymmetric BPE is differentiated from generalized BPE, which typically represents physiological enhancement rather than potential pathology. Lesion conspicuity was compared to BPE and rated as low, moderate, or high, where low refers to enhancement equal to or greater than BPE, high if enhancement was much greater than BPE, and moderate if enhancement was between low and high. Disagreements between readers were resolved by consensus. Two regions-of-interest (ROI), one representing the enhancing abnormality or BPE (characterized as focal asymmetric enhancement on both views), and the other non-enhancing background, respectively, were drawn by each radiologist on CEM recombined images for each patient to calculate: contrast enhancement (CE) and percent relative signal difference (%RSD), as measures of enhancement intensity. Lesion contrast enhancement was calculated for both the early (CC view; the contrast from the temporally early view [CE_early_]) and late (MLO view; the contrast from the temporally late view [CE_late_]) view, using CE = (*S*_a_ − *S*_b_)/*S*_b_, where *S*_a_ is the maximum pixel value within the lesion ROI, and *S*_b_ is the mean of the background ROI. %RSD was calculated as 100 × [(CE_late_ − CE_early_)/CE_early_] and used for the classification of CEM enhancement patterns as progressive (%RSD > 10%), plateau (−10% ≤ %RSD ≤ 10%), or wash-out (%RSD < −10%) [14]. Quantitative enhancement metrics (i.e., CE_early_, CE_late_, %RSD) for each lesion were calculated by averaging measurements by the readers. Lesion ROIs encompassed the entire enhancing abnormality. For background regions, care was taken to cover fatty components of the breast [13, 22], excluding glandular tissue or enhancing areas (Supplemental Fig. 1). Lesion size and enhancement type (mass or non-mass [18]) were additionally recorded.

#### MRI interpretation

MRI images were reviewed by at least two breast radiologists (> 5 years of experience) and findings were agreed by consensus with a panel of breast radiologists according to our departmental protocol. All images were reviewed with an evaluation of lesion morphology and enhancement kinetics determined using CADstream (Merge Healthcare Inc.).

A comparison of enhancement patterns between CEM and MRI was performed for a subgroup of patients with concurrent contrast-enhanced MRI as part of their clinical work-up (i.e., within 1–4 weeks of CEM).

### Histopathology

Lesions were histopathologically confirmed, with tumor histological subtype, hormone receptor, and lymph node status obtained from pathology reports. Benign lesions were verified by a 2-year follow-up. For analysis purposes, lesions were categorized as normal/benign (i.e., benign lesions and BPE) or malignant (i.e., invasive and non-invasive cancers).

### Statistical analysis

Statistical analysis was performed in jamovi, version 2.3 (The jamovi project, 2024; https://www.jamovi.org/) or GraphPad Prism, version 10.0.0 for Windows (GraphPad Software). Continuous data were assessed for normality using the Shapiro–Wilk test. Mann–Whitney *U*-test or Kruskal–Wallis test was used to compare two or more independent groups, respectively. Correlations between continuous variables were assessed using the Spearman correlation coefficient (ρ), while Kendall’s τ_b_ was used for correlations between ordinal, ordinal, and continuous variables. Categorical variable associations were evaluated using the chi-square (χ^2^) test. Results from post-hoc analyses were corrected for multiple comparisons using the Holm method. Interobserver agreement for conspicuity was assessed using Kendall’s coefficient of concordance (*W*), while Cohen’s κ was used to measure agreement between CEM and DCE-MRI enhancement patterns. The ability of quantitative conspicuity parameters (i.e., CE_early_, CE_late_, %RSD) in discriminating benign lesions or BPE from malignancy was assessed using receiver operating characteristic (ROC) curves and area under the curve (AUC). Classification thresholds for each parameter were determined using the maximum Youden index. Post-test probabilities for malignancy and benignity were calculated using Fagan nomograms. Statistical significance was defined as *p* < 0.05.

## Results

**Fig. 1:**
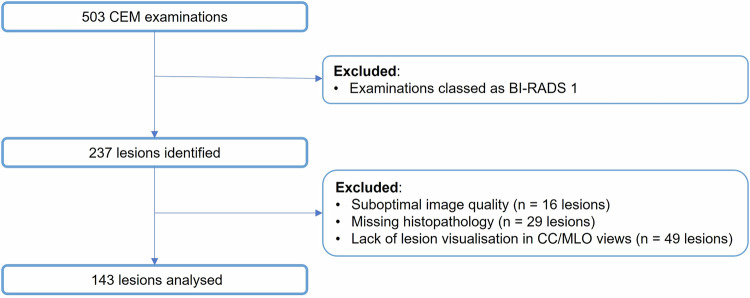
Study flowchart. CEM, contrast-enhanced mammography; BI-RADS, breast imaging reporting and data system; CC, craniocaudal; MLO, mediolateral oblique

A total of 237 BIRADS 2–5 lesions in 118 CEM examinations were selected retrospectively from 503 CEM studies. All lesions were biopsy-proven or followed up with mammography, CEM, and/or MRI for at least two years. Ninety-four out of two hundred thirty-seven (39.7%) lesions were excluded due to suboptimal image quality (*n* = 16/237, 6.8%), missing histopathology (*n* = 29/237; 12.2%), or lack of lesion visualization on both CC and MLO views (*n* = 49/237; 20.7%). The final cohort included 143 lesions (Fig. 1).

Of the lesions analyzed, 82/143 (57%) were classified as normal/benign either on biopsy or CEM/MRI at two-year follow-up (B1 or B2), 4/143 (3%) lesions were of uncertain malignant potential (B3) on biopsy, while the remaining 57/143 (40%) lesions were biopsy-proven cancers. Table 1 summarizes the clinicopathological characteristics of the patient population.

**Table 1:** Clinicopathological characteristics of the study population (*n* = 143 lesions)

Characteristic	*n* (%)
Age at diagnosis (years)^a^	58 [42–77]^a^
Histology (*n* = 143 lesions)	
Normal/benign	82 (57)
BPE (B1)	48 (33)
Benign (B2)^b^	34 (24)
Uncertain malignant potential (B3)^c^	4 (3)
Malignant	57 (40)
Invasive	49 (34)
Non-invasive	8 (6)
Tumor histology (*n* = 49 invasive lesions)	
No special type (NST)	36 (73)
Mixed^d^	3 (57)
Other^e^	10 (27)
Grade (*n* = 49 invasive lesions)	
1	8 (16)
2	28 (57)
3	13 (27)
Hormone receptor (HR) status (*n* = 49 invasive lesions)	
Negative	5 (88)
Positive	44 (12)
HER2 status (*n* = 49 invasive lesions)	
Negative	47 (96)
Positive	2 (4)
Molecular subtype (*n* = 49 invasive lesions)	
HR−/HER2−	5 (10)
HR+/HER2−	42 (86)
HR+/HER2+	2 (4)
Pathological lymph node status (*n* = 48 patients)	
Negative	23 (48)
Positive	25 (52)

*CEM* contrast-enhanced mammography, *HR* hormone receptor, *HER2* human epidermal growth factor 2

^a^ Data presented as median [range]

^b^ Benign lesions (*n* = 34): fibroadenoma (*n* = 12); fibrocystic and columnar cell change (*n* = 7); cyst (*n* = 6), benign intramammary node (*n* =  5); benign lesion on standard mammography/MRI (*n* = 2); duct ectasia (*n* = 1); and mastitis (*n* = 1)

^c^ Lesions of uncertain malignant potential (*n* = 4): fibrocystic and columnar cell change with lobular atypia (*n* = 1); intraductal papilloma without atypia (*n* = 1); cyst with radial scar (*n*  = 1); classical lobular carcinoma in situ (LCIS); and (*n* = 1)

^d^ Carcinomas involving the presence of two histological components (*n* = 3): NST + invasive lobular carcinoma (*n* = 2); NST + papillary carcinoma (*n* = 1)

^e^ Other histological subtypes (*n* = 10): invasive lobular carcinoma (*n* = 7); tubular carcinoma (*n* = 3)

### Qualitative assessment

**Fig. 2:**
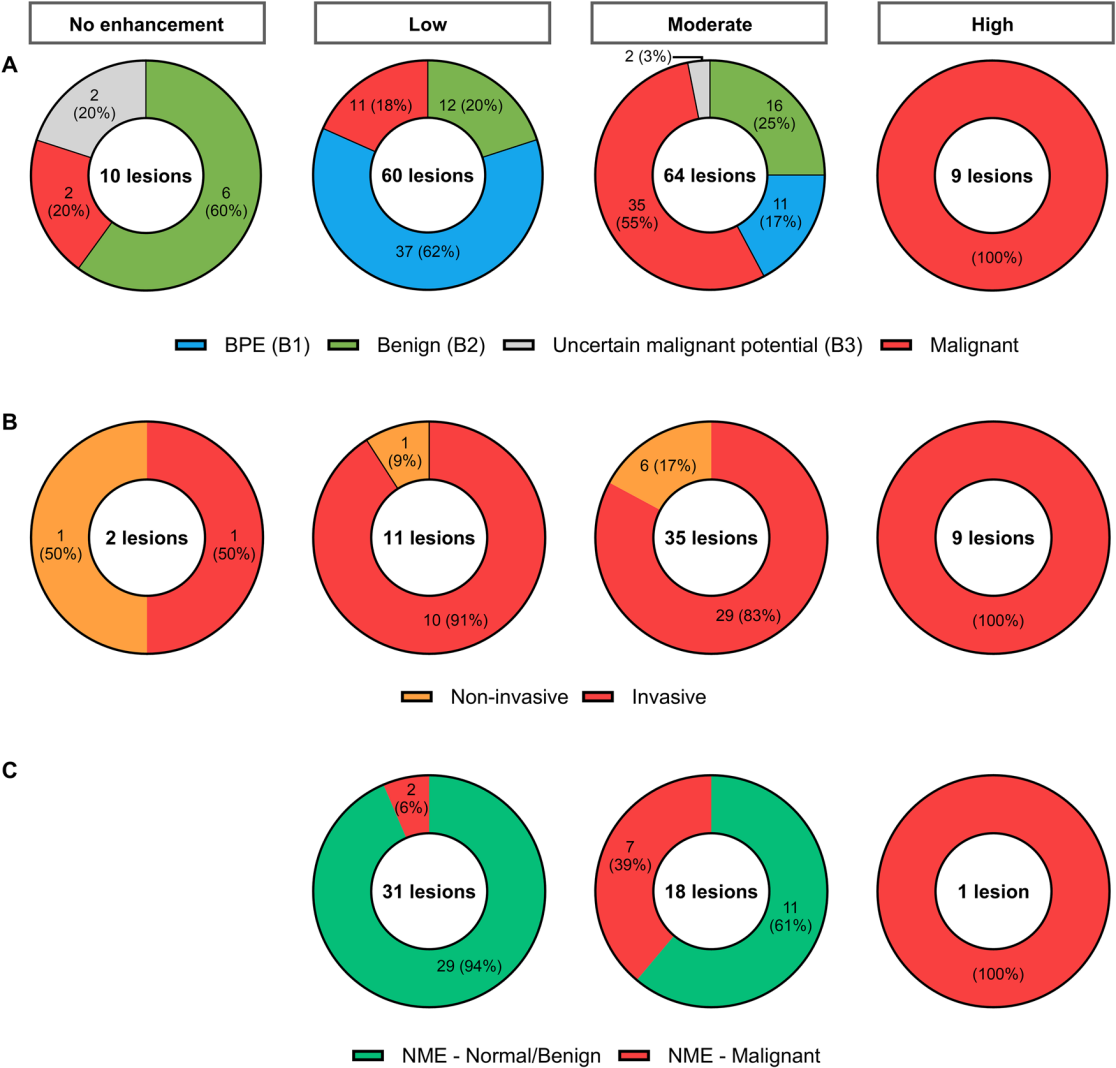
Lesion conspicuity (*n* = 143 lesions) with respect to **A** lesion histology **B** cancer type, and **C** normal/benign (B1/B2) or malignant lesions presenting as a non-mass enhancement (NME). Non-enhancing lesions included: B3 lesions—columnar and fibrocystic cell change with lobular atypia (*n* = 1), intraductal papilloma without atypia (*n* = 1); benign lesions—columnar cell and fibrocystic cell change without atypia (*n* = 2), cyst (*n* = 1), fibroadenoma (*n* = 1), mastitis (*n* = 1), benign-looking lesion on MRI (*n* = 1); malignant lesions—ductal carcinoma in situ (*n* = 1), tubular cancer, grade 1, HR+/HER2− (*n* = 1). The two B3 lesions showing moderate conspicuity in (**A**) included a classical lobular carcinoma in situ and a cyst with a radial scar. B1, normal histological diagnosis; B2, benign histological diagnosis; B3, uncertain malignant potential on histology; HR, hormone receptor; HER2, human epidermal growth factor 2

Qualitative assessment results are presented in Table 2. Lesion conspicuity was significantly associated with lesion histology (χ^2^ = 65.9, *p* < 0.001). Among low-conspicuity lesions, 49/60 (82%) were normal/benign, while 44/57 (77%) malignant lesions were either moderately or highly conspicuous (Fig. 2A). All high-conspicuity lesions (9/9, 100%) were invasive cancers. 6/8 (75%) non-invasive lesions were moderately conspicuous, while 1/8 (12.5%) lesions showed no enhancement (Fig. 2B). Additionally, 29/40 (71%) normal/benign lesions with non-mass enhancement showed low conspicuity (Fig. 2C). No significant association was observed between conspicuity and tumor grade (τ_b_ = −0.12, *p* = 0.36), molecular subtype (χ^2^ = 2.68, *p* = 0.26), or pathological lymph-node status (χ^2^ = 5.9, *p* = 0.12). However, all (9/9, 100%) high-conspicuity cases were intermediate/high-grade cancers of luminal subtype (HR-positive/HER2-negative) (Supplemental Table 1).

**Table 2:** Enhancement characteristics of lesions (*n* = 143) included the study

Characteristic	*n* (%)
Longest diameter (mm) on CEM^a^	14 [2–110]
Enhancement type (*n* = 143 lesions)	
Mass enhancement	83 (58)
Non-mass enhancement	50 (35)
No enhancement^b^	10 (7)
Conspicuity (*n* = 143 lesions)	
Low	60 (42)
Moderate	64 (45)
High	9 (6)
No enhancement^b^	10 (7)
Enhancement pattern (*n* = 143 lesions)	
Progressive	72 (50)
Plateau	38 (27)
Washout	23 (16)
No enhancement^b^	10 (7)

*CEM* contrast-enhanced mammography

^a^ Data presented as median [range]

^b^ Non-enhancing lesions included: B3 lesions—columnar and fibrocystic cell change with lobular atypia (*n* = 1), intraductal papilloma without atypia (*n* = 1); benign lesions—columnar cell and fibrocystic cell change without atypia (*n* = 2), cyst (*n* = 1), fibroadenoma (*n* = 1), mastitis (*n* = 1), benign-looking lesion on MRI (*n* = 1); malignant lesions—ductal carcinoma in situ (*n* = 1), tubular cancer, grade 1, HR+/HER2− (*n* = 1)

Conspicuity assessment demonstrated strong interobserver agreement (*W* = 0.87, *p* < 0.001), with complete agreement (*W* = 1) for B3 (*n* = 4) and non-enhancing (*n* = 10) lesions. Reader agreement (Kendall’s *W*) was 0.55, 0.87, and 0.88 for BPE, benign and malignant lesions, respectively.

**Fig. 3:**
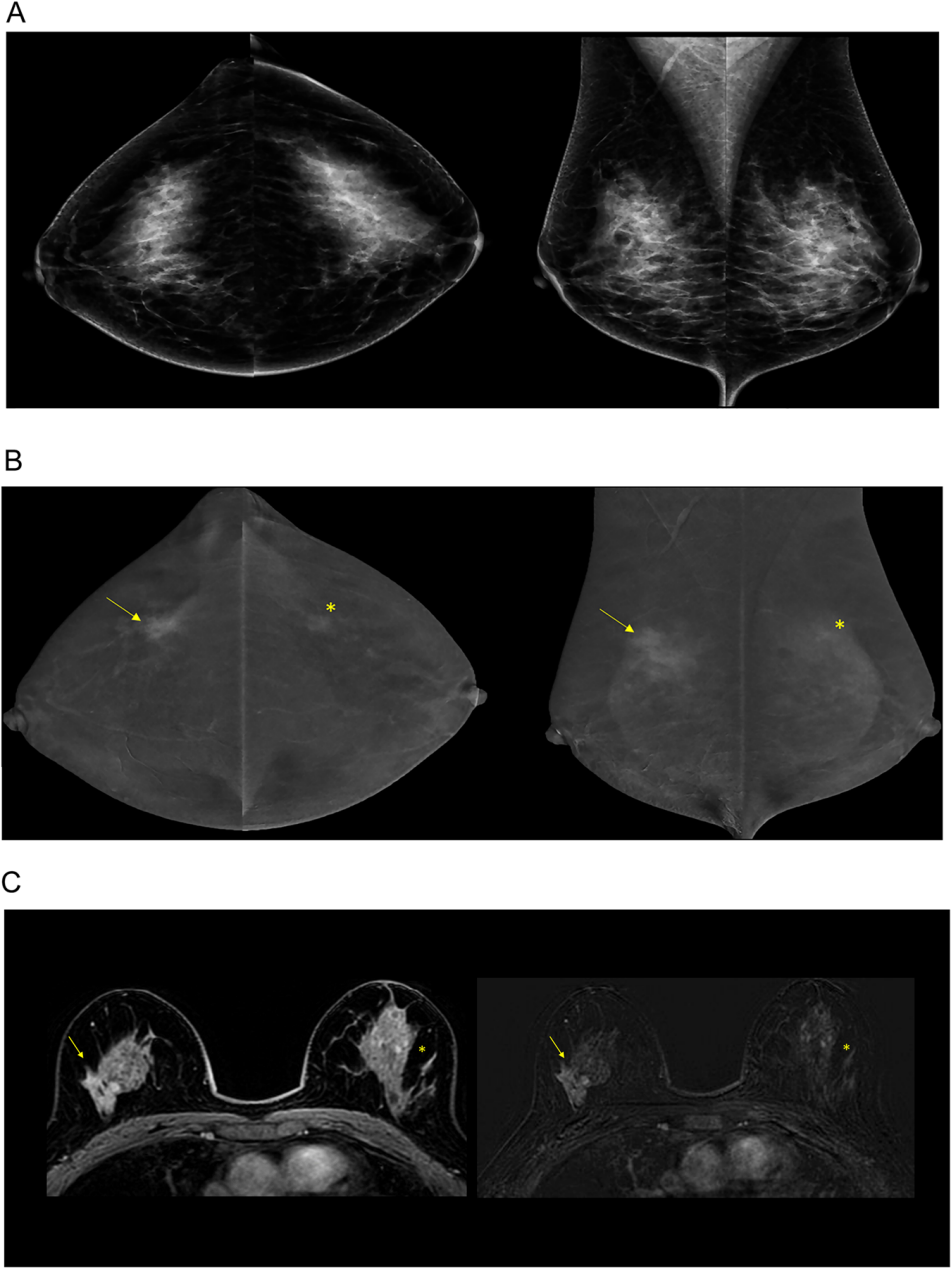
Representative CEM and MR images of a 51-year-old female from the BRAID trial with normal screening mammograms and lesions of moderate and low conspicuity on CEM. **A** low-energy mammograms in CC (left) and MLO (right) views; **B** CEM recombined images in CC (left) and MLO (right) views demonstrating asymmetric non-mass enhancement (NME) of moderate conspicuity in the right upper outer quadrant (yellow arrows in the right breast, CE_early_: 0.059, CE_late_: 0.054, %RSD: −5.6%). Histopathology confirmed an invasive NST carcinoma, grade 1, HR+/HER2− with intermediate and high-grade DCIS on biopsy. NME with low conspicuity in the left upper outer quadrant (asterisk in the left breast, CE_early_: 0.038, C_Eate_: 0.05, %RSD: 23%) in keeping with BPE, as confirmed by contrast-enhanced breast MRI; **C** corresponding post-contrast axial MRI and subtraction images showing the asymmetric NME in the upper quadrant of the right breast (yellow arrows). The punctate enhancement in the left breast was related to BPE (yellow asterisks). CEM, contrast-enhanced mammography; CC, craniocaudal; MLO, mediolateral oblique; CE_early_, contrast from the early view; CE_late_, contrast from the late view; %RSD, relative signal difference; NST, no special type; DCIS, ductal carcinoma in situ; HR, hormone receptor; HER2, human epidermal growth factor 2

Figure 3 shows representative CEM low-energy and recombined images, alongside the corresponding MRI postcontrast images, illustrating lesions with low and moderate conspicuity.

### Quantitative assessment

**Fig. 4:**
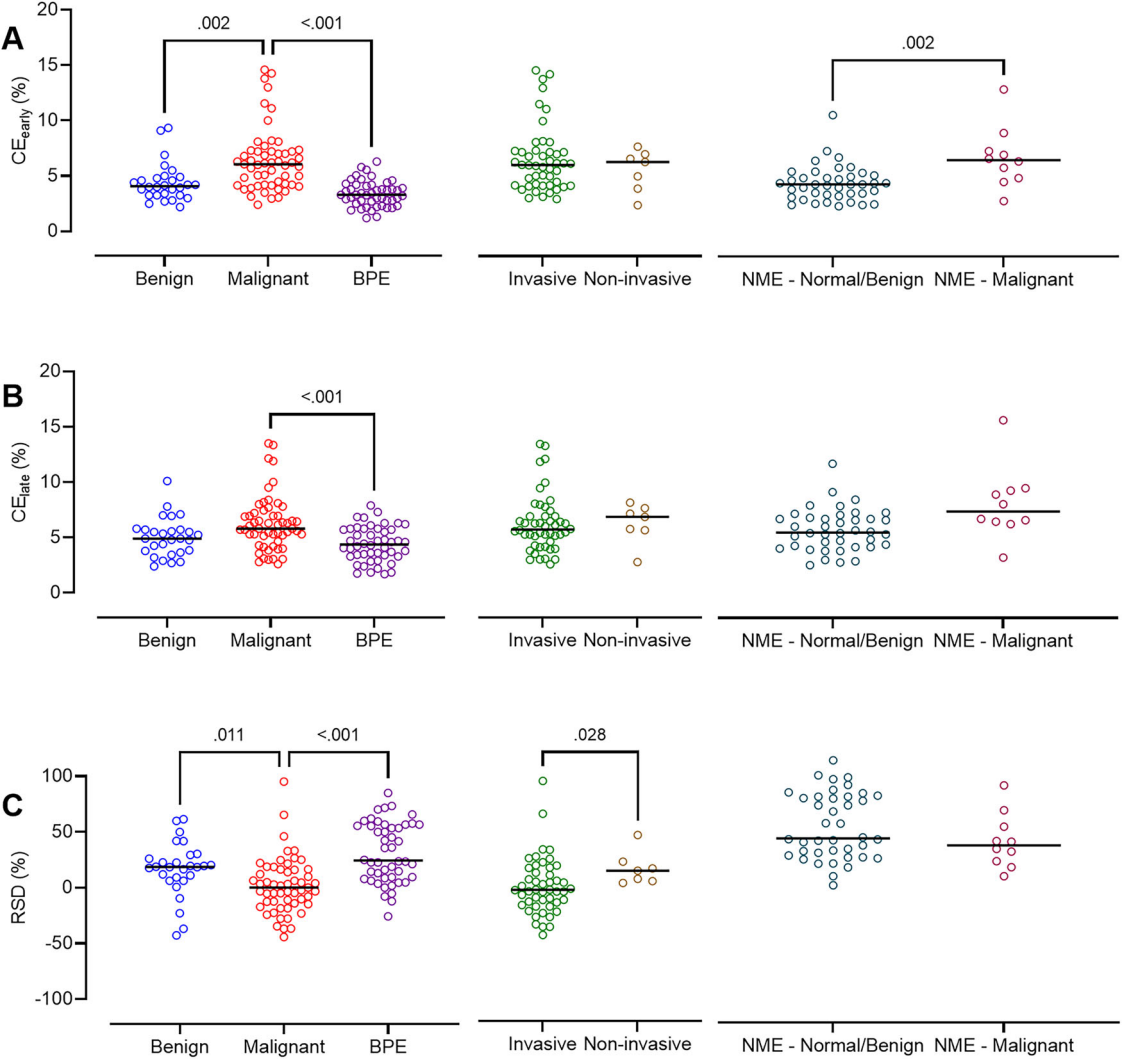
Scatter dot plots of (**A**) contrast ratio from the temporally early view (CE_early_:), **B** contrast from the temporally late view (CE_late_), and **C** percent residual signal difference (%RSD) in (left) benign (*n* = 28) vs malignant lesions (*n* = 55), or BPE (*n* = 48); (middle) invasive (*n* = 49) vs non-invasive cancers (*n* = 7); (right) normal/benign (*n* = 40) vs malignant (*n* = 10) lesions presenting as a non-mass enhancement (NME). Non-enhancing lesions and/or those of uncertain malignant potential on histology (*n* = 12) were excluded from this analysis. The black line indicates the median. Non-significant *p*-values are not shown in the figure. CE_early_, invasive vs non-invasive, *p* = 0.71; CE_late_, invasive vs non-invasive, *p* = 0.45; CE_late_, NME-normal/benign vs NME-malignant, *p* = 0.05; %RSD: NME-Normal/benign vs NME-malignant, *p* = 0.2. BPE, background parenchymal enhancement; B1, normal histology; B2, benign histology

Lesion conspicuity significantly correlated with CE_early_ (τ_b_ = 0.52, *p* < 0.001), CE_late_ (τ_b_ = 0.52, *p* < 0.001), or %RSD (τ_b_ = −0.23, *p* < 0.001). Both CE_early_ and CE_late_ were significantly lower for benign lesions or BPE than malignancy, with no significant difference between invasive and non-invasive cancers (Fig. 4A, B). Lower CE_early_ values were observed in non-mass enhancing normal/benign than malignant lesions (Fig. 4A). In contrast, %RSD was higher in benign lesions or BPE than in malignancy, and non-invasive vs invasive cancers (Fig. 4C). No significant difference in quantitative metrics was observed between BPE and benign lesions.

### CEM enhancement patterns

CEM enhancement patterns are significantly associated with lesion conspicuity (χ^2^ = 12.6, *p* = 0.01). Low-conspicuity lesions predominantly exhibited progressive enhancement (39/60; 65%), while 7/9 (78%) highly conspicuous lesions demonstrated wash-out or plateau enhancement (Supplemental Fig. 2). Lesion histology was associated with enhancement pattern (χ^2^ = 48.1, *p* < 0.001), with 54/82 (66%) normal/benign lesions exhibiting progressive enhancement, while 38/57 (67%) cancers showed plateau/wash-out patterns (Supplemental Fig. 2). Cancer type was not significantly associated with CEM enhancement pattern (χ^2^ = 6.4, *p* = 0.1), although invasive tumors more frequently demonstrated plateau/wash-out enhancement (38/49; 78%), and 7/8 (87.5%) non-invasive cancers showed progressive/plateau enhancement (Supplemental Fig. 2). CEM enhancement type was not significantly associated with tumor grade (χ^2^ = 7.8, *p* = 0.25), molecular subtype (χ^2^ = 3.3, *p* = 0.51) or lymph-node status (χ^2^ = 2.7, *p* = 0.44).

### Agreement between CEM and DCE-MRI enhancement patterns

**Fig. 5:**
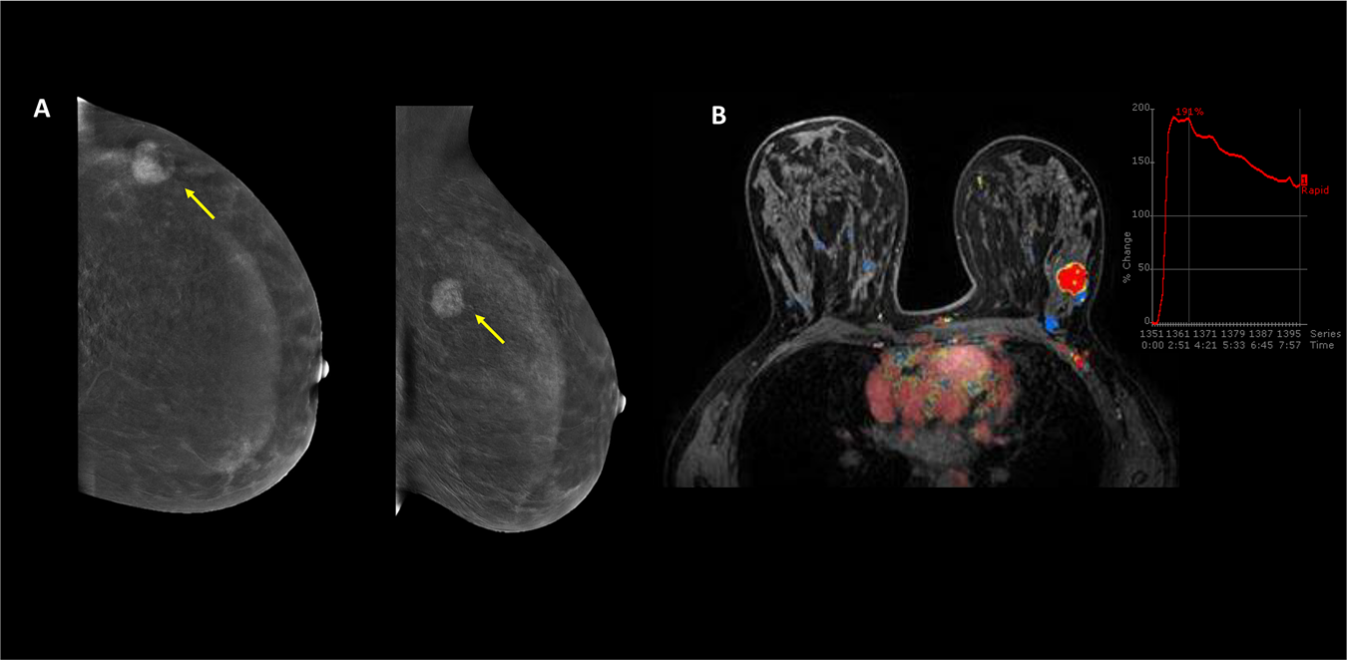
Representative CEM recombined and MR images for a 38-year-old woman with invasive NST carcinoma, grade 3, HR+/HER2− and high-grade DCIS. **A** Enhancing mass lesion in the left upper outer quadrant (yellow arrows) demonstrating high conspicuity on CC and MLO views and wash-out enhancement pattern (%RSD: −22.5). **B** Corresponding post-contrast MRI with type III enhancement. CEM, contrast-enhanced mammography; NST, no special type; HR, hormone receptor; HER2, human epidermal growth factor 2; DCIS, ductal carcinoma in situ; CC, craniocaudal; MLO, mediolateral oblique; %RSD, percent relative signal difference

In a subgroup of 66 lesions (Supplemental Table 2) with concurrent DCE-MRI (median interval between CEM and MRI, 17 days [IQR: 10, 30]), agreement between CEM and MRI enhancement types was fair-moderate (Cohen’s κ = 0.35, *p* < 0.001), with 5/8 (63%) lesions lacking enhancement on CEM showing progressive patterns on MRI. The degree of agreement was higher for lesions exhibiting a wash-out pattern (Cohen’s κ = 0.5, *p* < 0.001) than those with progressive (Cohen’s κ = 0.30, *p* = 0.01) or plateau enhancement (Cohen’s κ = 0.31, *p* = 0.01). Figure 5 illustrates example CEM images of a highly conspicuous malignant lesion with wash-out enhancement, alongside the corresponding post-contrast MRI.

### Diagnostic performance of qualitative and quantitative descriptors of conspicuity for differentiating benign from malignant lesions

**Fig. 6:**
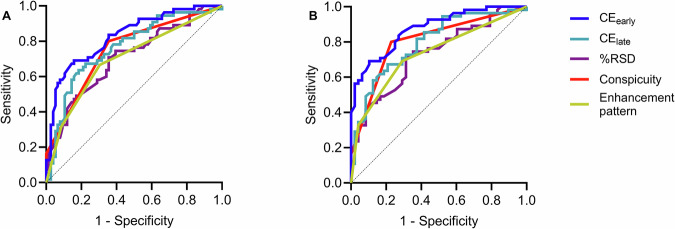
Receiver operating characteristic (ROC) curves for differentiating **A** benign vs malignant lesions, **B** BPE vs malignant lesions. A total of 131 lesions were included in this analysis. Non-enhancing lesions and/or those of uncertain malignant potential on histology (*n* = 12) were excluded

Tables 3 and 4 summarize the diagnostic performance of qualitative and quantitative descriptors of conspicuity, together with classification thresholds for distinguishing malignancy from normal/benign lesions or BPE. Figure 6 illustrates ROC curves for all conspicuity metrics. CE_early_ performed better in differentiating normal/benign lesions or BPE from cancers compared to other metrics, with post-test probabilities of malignancy at 76% and 88%, respectively (Tables 2 and 3).

**Table 3:** Diagnostic performance of conspicuity descriptors and classification thresholds for differentiating benign (*n* = 28) from malignant (*n* = 55) lesions

	Conspicuity metrics				
	Conspicuity	Enhancement pattern	CE_early_	CE_late_	%RSD
AUC [95% CI]	0.75 [0.67–0.84]	0.72 [0.63–0.80]	0.83 [0.76–0.90]	0.71 [0.62–0.80]	0.77 [0.68–0.85]
Accuracy (%)	71	70	78	67	74
Sensitivity (%)	80	66	84	71	64
Specificity (%)	65	73	77	65	82
Classification threshold	Moderate	Plateau	4.8	5.3	5.5
Post-test probability of malignancy (%)	62	62	76	59	71
Post-test probability of benignity (%)	81	76	79	75	75

The analysis involved enhancing lesions, excluding non-enhancing lesions and/or those of uncertain malignant potential on histology (*n* = 12)

*AUC* area under the curve, *CI* confidence interval, *CE*_*early*_ contrast enhancement in the temporally early recombined view, *CE*_*late*_ contrast in the recombined temporally late view, *%RSD* percent relative signal difference

**Table 4:** Diagnostic performance of conspicuity descriptors and classification thresholds for differentiating BPE (*n* = 48) from malignant lesions (*n* = 55)

	Conspicuity metrics				
	Conspicuity	Enhancement pattern	CE_early_	CE_late_	%RSD
AUC [95% CI]	0.80 [0.72–0.89]	0.73 [0.64–0.83]	0.88 [0.81–0.94]	0.73 [0.64–0.83]	0.77 [0.71–0.88]
Accuracy (%)	79	70	79	70	78
Sensitivity (%)	80	71	69	71	67
Specificity (%)	77	69	90	69	79
Classification threshold	Moderate	Plateau	4.8	5.3	7.2
Post-test probability of malignancy (%)	62	62	88	72	79
Post-test probability of benignity (%)	81	76	71	67	68

The analysis involved enhancing lesions, excluding non-enhancing lesions and/or those of uncertain malignant potential on histology (*n* = 12)

*AUC* area under the curve, *CI* confidence interval, *CE*_*early*_ contrast-to-noise ratio in the temporally early view, *CE*_*late*_ contrast in the temporally late view, *%RSD* percent relative signal difference

## Discussion

This retrospective analysis of selected lesions from screening and symptomatic CEM research studies suggests that quantifying lesion enhancement using contrast can enhance CEM specificity and overall diagnostic performance in differentiating benign lesions or BPE from malignancy. Lesion contrast from the early recombined view outperformed other metrics in differentiating malignancy from benign lesions or BPE, with AUC values of 0.83 and 0.88, respectively, and a specificity of 90% when discriminating malignant lesions from BPE.

CEM is an emerging contrast-enhanced imaging technique, with high diagnostic sensitivity but relatively low specificity [22]. Its growing utilization in diagnostic and screening settings necessitates additional methods to improve specificity and reduce unnecessary recalls [23]. Previous studies have investigated qualitative and quantitative lesion enhancement on CEM for predicting malignancy with variable results [13, 14, 24–26]. To our knowledge, this is the first comprehensive study analyzing conspicuity in relation to quantitative metrics of lesion enhancement on CEM recombined images and comparing their diagnostic performance.

Conspicuity is a novel descriptor introduced in the recent BI-RADS CEM lexicon that could potentially enhance lesion characterization. Recent studies suggest that lesion conspicuity on CEM demonstrates satisfactory diagnostic performance in predicting malignancy, with high conspicuity significantly correlating with ER/PR-negative or grade 3 cancers [19], while the low conspicuity of benign lesions on CEM has been found to potentially help reduce false positives in clinical practice [27]. Similarly, our study revealed a significant correlation between conspicuity and lesion histology, with most low-conspicuous lesions being normal/benign, and malignant lesions showing predominantly high conspicuity. Although the limited number of cancers in our study may explain the lack of a significant association between conspicuity and tumor histopathology, our results are broadly consistent with those of previous studies [19, 28], which also reported non-significant associations between conspicuity and cancer histopathology, along with a significant positive correlation between conspicuity and cancer grade [19].

Conspicuity assessment showed strong interobserver agreement (*W* = 0.87), with full consensus for B3 and non-enhancing lesions. However, reader agreement was lower for BPE (*W* = 0.55) than benign (*W* = 0.87) or malignant lesions (*W* = 0.88). The moderate inter-reader agreement regarding BPE conspicuity likely arises from differing perceptions of mild and moderate BPE among readers, consistent with observations from a previous study on BPE classification on CEM [29].

We observed a significant correlation between lesion histology and contrast enhancement from both the early and late recombined views. Despite methodological differences in the quantitative assessment of CEM enhancement between published studies, our findings align with those of previous reports [6, 13, 14, 24, 30], showing significantly higher signal enhancement in malignant than benign lesions. The contrast from the early recombined view (CE_early_) allowed better differentiation between benign and malignant lesions, indicating that lesion enhancement measured within 3 min after contrast administration can provide important information about malignancy. However, unlike Liu et al [13], quantitative metrics or CEM enhancement type were not significantly associated with tumor histopathology. This may be attributed to the limited number of cancers in our study, mostly hormone receptor-positive ductal carcinomas.

Lesion histology was significantly associated with CEM enhancement pattern, with normal/benign lesions exhibiting progressive enhancement, while cancers predominantly followed plateau or wash-out patterns. This observation is in agreement with Deng et al [14], who reported an association between decreasing enhancement patterns on CEM and malignancy and is consistent with DCE-MRI results, where wash-out enhancement typically indicates malignancy [31, 32].

Consistent with Liu et al [13], we found non-significant differences in quantitative metrics between invasive and non-invasive cancers, with 56% of non-invasive cancers showing progressive enhancement. However, there is conflicting literature regarding the differentiation between invasive and non-invasive diseases using CEM. Rudnicki et al [24] found significant differences in quantitative metrics between invasive and in situ carcinoma, while Liu et al [13] noted differences in enhancement intensity between benign and in situ disease, but not between non-invasive and infiltrating cancers. Our findings mirror observations from contrast-enhanced MRI, where approximately half of the non-invasive lesions exhibit persistent enhancement [33, 34].

Despite insufficient data on the diagnostic performance of CEM, previous studies indicate high sensitivity and moderate specificity that is comparable to MRI [35, 36]. In our study, CE_early_ showed superior performance in differentiating normal/benign from malignant lesions (AUC = 0.83) than other metrics, in agreement with reported AUC values (0.70–0.88) in previous studies [13, 19, 24, 25]. Furthermore, we could differentiate between BPE and cancers with a specificity of 90%. Our results broadly agree with those of Boca et al, who used CEM-based radiomics to differentiate BPE from malignancy [37].

In a subgroup of 66 lesions, the agreement between DCE-MRI and CEM enhancement types was fair-moderate (Cohen’s κ = 0.35), which is lower than the value reported in Liu et al (Cohen’s κ = 0.52) [13]. This difference was possibly related to the smaller sample size (*n* = 28) investigated with MRI in the latter study, which included only symptomatic cases. Interestingly, we observed higher agreement between CEM and DCE-MRI enhancement patterns for lesions exhibiting wash-out enhancement (Cohen’s κ = 0.5) than those with progressive (Cohen’s κ = 0.30) or plateau enhancement (Cohen’s κ = 0.31).

Similar to a previous study [13], we utilized the maximum value within the lesion for contrast enhancement calculations rather than the average, as the former is independent of region placement, lesion shape, and size, thereby providing a consistent and reproducible approach for lesion enhancement quantification. Furthermore, the maximum value better represents peak contrast in heterogeneous lesions, although it is more susceptible to noise. While the average would better reflect overall lesion intensity, it is also more significantly affected by partial volume effects and region-boundary inconsistencies.

Our study has several limitations. First, it is a single-center retrospective study with a limited number of cancers and benign lesions. Second, the lack of standardization in contrast injection times and dose are known limitations of CEM that might affect the quantification of enhancement intensity and patterns [38, 39]. Third, our investigation focused exclusively on assessing lesion enhancement on recombined images, without taking into consideration features such as shape, margins, calcifications, or distortion in low-energy images. Given the significant role of low-energy images in the evaluation of CEM in clinical practice, combining features from low-energy mammograms with the degree of enhancement, along with consensus reading for discordant cases, could lead to more accurate results [40]. Fourth, approximately 40% of lesions were excluded at initial screening due to issues such as the lack of lesion visualization on CC and MLO views, suboptimal image quality, or missing histopathology. While these exclusions may have affected the representativeness of our lesion sample, applying these exclusion criteria was necessary to maintain the accuracy and reliability of our results. Lastly, the retrospective nature of our investigation prevented us from evaluating the effect of differences in lesion configuration between CC and MLO views on contrast measurements and RSD at individual time points. However, a previous study found non-significant differences in signal enhancement and contrast between the two views [22]. Additionally, our methodology for contrast determination was comparable between the two views.

In conclusion, our results indicate that quantifying lesion enhancement conspicuity and patterns can improve CEM specificity and diagnostic performance in differentiating BPE or benign lesions from malignancy, potentially reducing unnecessary recalls and benign biopsies. Incorporating lesion conspicuity into routine CEM reporting could enhance lesion evaluation and diagnostic accuracy. Standardization of CEM protocols combined with AI-assisted tools for prompt lesion delineation and enhancement quantification may boost robustness and streamline clinical implementation [41]. Large multicenter studies are needed to establish reliable conspicuity thresholds and validate their integration with AI tools to optimize clinical workflows.

## Supplementary information

ELECTRONIC SUPPLEMENTARY MATERIAL

## Abbreviations

BPE: Background parenchymal enhancement
CE_early_: Contrast from the temporally early view
CE_late_: Contrast from the temporally late view
CEM: Contrast-enhanced mammography
RSD: Relative signal difference
