# Supplementary material for: Improving the diagnostic performance of contrast-enhanced mammography through lesion conspicuity and enhancement quantification
Source: Eur Radiol. 2025 Apr 3;35(10):6385–97. doi: 10.1007/s00330-025-11501-8 (PMC12417241; doi:10.1007/s00330-025-11501-8)
Supplement: Supplementary file 1 — ELECTRONIC SUPPLEMENTARY MATERIAL [file 330_2025_11501_MOESM1_ESM.pdf]

Improving the diagnostic performance of Contrast-Enhanced Mammography through lesion conspicuity and enhancement quantification

ELECTRONIC SUPPLEMENTARY MATERIAL

A B

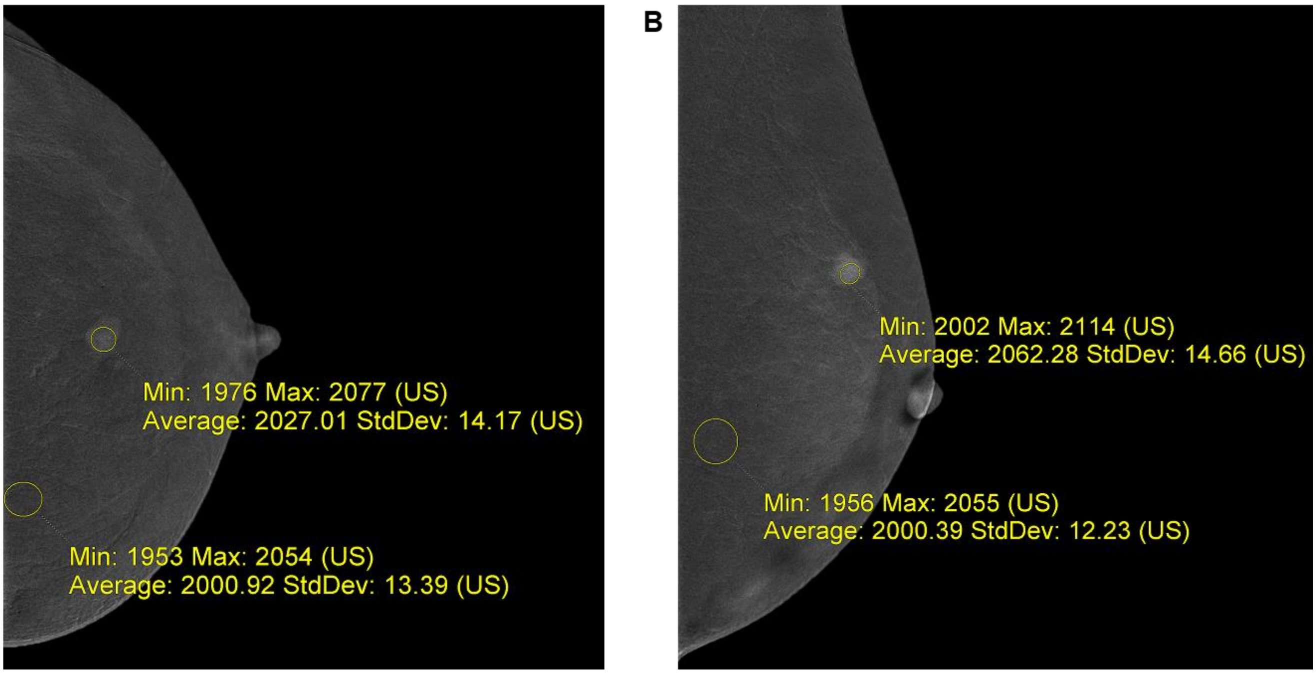

Supplemental Figure 1: Signal enhancement quantification on CEM recombined images: two circular regions representing the enhancing lesion and background, respectively, were placed on the craniocaudal (CC) and medial-lateral oblique (MLO) views in each patient examination. Both views were analyzed using comparable methodology, with similar-sized lesions on each view for the background and each lesion. The maximum value within lesion ROIs was used for contrast calculations. CEM: contrast-enhanced mammography; ROI: region of interest.

Supplemental Table 1: Conspicuity metrics with respect to tumor histopathology.

Characteristic	n (%)	Conspicuity			
		No enhancement	Low	Moderate	High
All cancers	49	1	10	29	9
Histological subtype					
NST	36 (74)	-	9	19	8
Mixed	3 (6)	-	1	2	-
ILC	7 (14)	-	-	6	1
Tubular	3 (6)	1	-	2	
Grade					
1	8 (16)	1	-	7	-
2	28 (57)	-	4	17	7
3	13 (27)	-	6	5	2
Molecular subtype					
HR-/HER2-	6 (12)	-	2	3	1
HR+/HER2-	40	1	7	25	8
HR+/HER2+	2	-	1	1	-
Lymph node status[a]					
Negative	23 (48)	1	2	18	2
Positive	25 (52)	1	7	11	8

[a] In n=48 patients.
NST: no specific type; Mixed: cancers with two histological components; HR: hormone receptor; HER2: human epidermal growth factor 2.

Supplemental Table 2: Percentage of normal/benign lesions assessed through biopsy or follow-up

**Characteristic**	***n* (%)**
Benign lesions (*n* = 34 lesions)	
Biopsy	25 (73.5)
Follow-up	9 (26.5)
Background parenchymal enhancement (*n* = 48 lesions)	
Biopsy	10 (20.8)
Follow-up	38 (79.2)

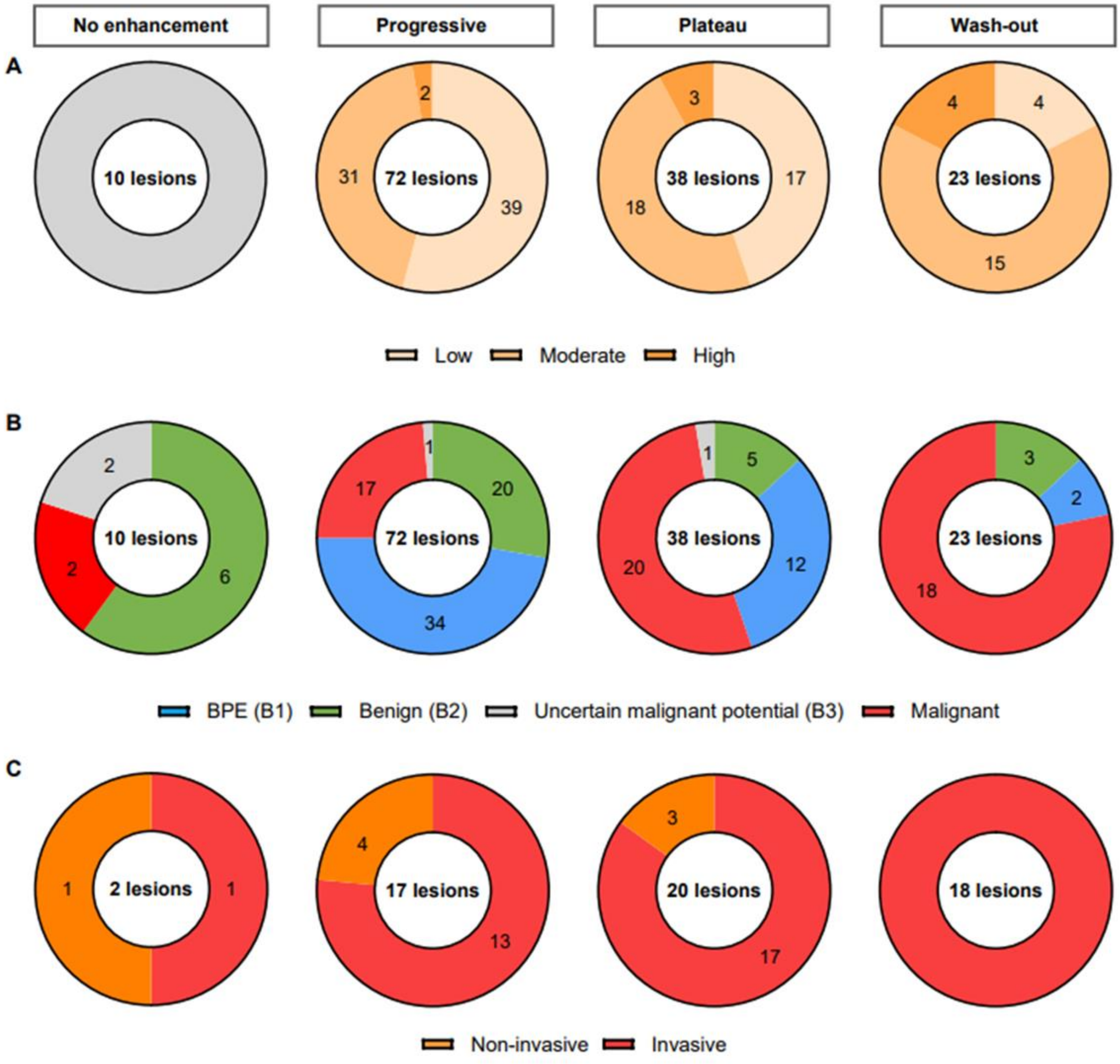

Supplemental Figure 2: CEM enhancement patterns with respect to (**A**) lesion conspicuity (**B**) lesion histology, and (**C**) cancer type.
CEM: contrast-enhanced mammography; BPE: background parenchymal enhancement; B1: normal histological diagnosis; B2: benign on histological diagnosis; B3: uncertain malignant potential on biopsy.

Supplemental Table 3: Histological characteristics of lesions (n=66) with concurrent contrast-enhanced MRI and CEM.

**Characteristic**	***n* (%)**
Histology (*n* = 66 lesions)	
Normal/benign	27 (41)
Background parenchymal enhancement (B1)	14 (21)
Benign (B2)	13 (20)
Uncertain malignant potential (B3)	3 (4.5)
Malignant	36 (54.5)
Invasive	32 (48.5)
Non-invasive	4 (6)
